# Adiponectin promotes human jaw bone marrow mesenchymal stem cell chemotaxis *via* CXCL1 and CXCL8

**DOI:** 10.1111/jcmm.13070

**Published:** 2017-02-08

**Authors:** Yinfei Pu, Mengke Wang, Yingying Hong, Yuwei Wu, Zhihui Tang

**Affiliations:** ^1^ 2nd Dental CenterPeking University School and Hospital of StomatologyBeijingChina; ^2^ Central LaboratoryPeking University School and Hospital of StomatologyBeijingChina; ^3^ Department of PathologyPeking University School and Hospital of StomatologyBeijingChina; ^4^ National Engineering Laboratory for Digital and Material Technology of StomatologyPeking University School and Hospital of StomatologyBeijingChina

**Keywords:** adiponectin, jaw bone, stem cells, chemotaxis, CXCL1, CXCL8

## Abstract

Adiponectin (APN) is known to promote the osteogenic differentiation of human jaw bone marrow mesenchymal stem cells (h‐JBMMSCs). However, the underlying mechanism has not been fully elucidated. Previously, we showed that APN could promote h‐JBMMSC osteogenesis *via* APPL1‐p38 by up‐regulating osteogenesis‐related genes. Here, we aimed to determine whether APN could promote h‐JBMMSC chemotaxis through CXCL1/CXCL8. The CCK‐8, wound healing and transwell assays were used to evaluate the proliferation, migration and chemotaxis of h‐JBMMSCs with or without APN treatment. Chemotaxis‐related genes were screened using RNA‐seq, and the results were validated using real‐time PCR and ELISA. We also performed Western blot using the AMPK inhibitor, WZ4003, and the p38 MAPK inhibitor, SB203580, to identify the signalling pathway involved. We found that APN could promote h‐JBMMSC chemotaxis in the co‐culture transwell system. CXCL1 and CXCL8 were screened and confirmed as the up‐regulated target genes. The APN‐induced CXCL1/8 up‐regulation to promote chemotaxis could be blocked by CXCR2 inhibitor SB225002. Western blot revealed that the phosphorylation of AMPK and p38 MAPK increased in a time‐dependent manner with APN treatment. Additionally, WZ4003 and SB203580 could suppress the APN‐induced overexpression of CXCL1 and CXCL8. The results of the transwell chemotaxis assay also supported the above results. Our data suggest that APN can promote h‐JBMMSC chemotaxis by up‐regulating CXCL1 and CXCL8.

## Introduction

Adiponectin was discovered in 1995 and is a 30‐kD protein that contains a globular domain (residues 111–247) 1. The functions of this globular domain are similar to those of the full‐length APN, and its structure is easy to investigate 2. Both the globular domain of APN and the full‐length APN can stimulate 5′AMP‐activated protein kinase (AMPK) activity 3. In addition, both of them can bind the APN receptors, AdipoR1 and AdipoR2 4, and the interaction between them and the receptors proceeds between the globular domain of APN and the extracellular surface of the receptors 2.

APN exerts various physiological effects on many types of cells by suppressing cell death and inflammation and enhancing cell survival 2. Recombinant APN is known to be able to suppress the apoptosis of human umbilical vein endothelial cells 5 and auditory HEI‐OC1 cells 6. APN also protects against ozone‐induced lung inflammation 7 and inhibits TNFα‐induced inflammatory reactions 8 in animal models. Moreover, APN can activate many signalling pathways such as the AMPK and peroxisome proliferator‐activated receptor (PPAR)‐alpha signalling pathways through its interaction with its receptors, AdipoR1 and AdipoR2 9, 10. The adaptor protein for APN contains a pleckstrin homology domain, phosphotyrosine binding domain and leucine zipper motif (APPL1) and interacts with both AdipoR1 and AdipoR2 to mediate the downstream effects of APN on AMPK activation, glucose uptake and lipid oxidation 11. APN can also up‐regulate myocyte enhancer factor‐2 by activating p38 MAPK phosphorylation in primary cardiomyocytes 12. APN has also been shown to promote the migration of BMSCs from the bone marrow into the circulation to regenerate bone by regulating stromal cell‐derived factor‐1 expression in an animal model 13. APN is known to increase CXCL1 expression in tumour cells to promote stromal cell senescence 14.

CXCL1 belongs to the chemokine family, along with CXCL8 and many others 15. CXCL1 binds to CXCR2, and CXCL8 binds to CXCR1 as well as CXCR2 specifically 16. CXCL1 and CXCL8 have been reported to bind with CXC receptors to form the CXCL/CXCR ligand/receptor complex, which activates downstream signalling pathways related to cell migration in tissue engineering studies 17, 18. The CXCL1–CXCR2 axis is known to play a role in the recruitment of bone marrow‐derived endothelial progenitor cells to the lung 19 and leucocytes in venules 20. CXCL8 can also promote the migration of periosteal progenitor cells in a dose‐dependent manner 21. The CXCL8‐CXCR1/CXCR2 axis has also been shown to increase the migratory ability of bone marrow mesenchymal stem cells (BMSCs) in a dose‐dependent manner 22.

In our previous study, we showed that APN could promote the osteogenesis of extraction sockets of beagle dogs *in vivo* 23. In another *in vitro* study, APN was found to promote the osteogenesis of human jaw bone mesenchymal stem cells (h‐JBMMSCs) through the APPL1‐p38 MAPK signalling pathway 24. Bone regeneration in response to bioactive molecules requires the recruitment, proliferation and osteogenic differentiation of mesenchymal progenitors 25. Based on these data, we suggested that APN promotes the direct migration of h‐JBMMSCs to extraction sockets during APN‐induced osteogenesis. Therefore, in this study, we screened two target genes CXCL1 and CXCL8 in h‐JBMMSCs using RNA‐seq sequencing with or without APN treatment to determine whether CXCL1 and CXCL8 played a role in APN‐induced chemotaxis. We also then attempted to identify the signalling pathway involved in this process.

## Materials and methods

### Cells and cell culture

The h‐JBMMSCs were cultured as described in our previous study, and the protocol was approved by the Medical Ethical Commission of the Peking University School of Stomatology (PKUSSIRB‐201520026). The informed consent was obtained from all the patients. The h‐JBMMSCs were obtained from patients undergoing orthognathic surgery and were identified based on positivity for CD73, CD90 and CD105 and negativity for CD34, CD11b, CD19, CD45 and HLA‐DR using flow cytometry 23, 24. The osteoblast‐inducing conditional medium consisted of α‐MEM medium with 10% FBS, 10 nM dexamethasone, 10 mM β‐glycerophosphate and 50 μg/ml L‐ascorbic acid (Sigma‐Aldrich, St. Louis, MO, USA). The complete medium consisted of α‐MEM medium with only 10% FBS. The cells were cultured with or without 1 μg/ml APN (dissolved in PBS, Z03072; GenScipt, Piscataway, NJ, USA) produced using HEK 293 cells with endotoxin <0.01 EU/μg (determined using the LAL method; data not shown). For the CXCR2 inhibitor (10 μM working concentration dissolved in DMSO, SB225002; Selleck, Boston, MA, USA), p38 inhibitor (10 μM working concentration dissolved in DMSO, SB203580; Selleck, USA) and AMPK inhibitor (5 μM working concentration dissolved in DMSO, WZ4003; Selleck, USA) assay, the cells were treated with the inhibitors for 2 hrs before the experiments.

### Cell proliferation assay

Cell proliferation was measured *in vitro* using the CCK‐8 assay (Dojindo, Tokyo, Japan). Briefly, 3 × 10^3^ h‐JBMMSCs were plated onto 96‐well plates with 100 μl medium per well. Following overnight incubation, 10 μl of CCK‐8 solution was added to ten wells and incubated for 2 hrs in the incubator. Colour evaluation was performed photometrically at 450 nm using an ELx808 absorbance microplate reader (BioTeK Instruments, Winooski, VT, USA). All the cells were cultured with complete medium (data not shown) or osteoblast‐inducing conditional medium for 8 days to determine the proliferation rate. During these 8 days of culture, PBS and APN were added to the control group and APN‐treatment group, respectively, every 3 days.

### Wound healing assay

A total of 5 × 10^5^ h‐JBMMSCs was cultured as confluent monolayers and wounded by scratching across the 6‐well plates with a 200‐μl pipette tip. The deciduous cells were removed using PBS. The wounded monolayers were then photographed at 10× magnification (Nikon, Tokyo, Japan) and analysed 0, 6 and 12 hrs after scratching. Three different media were applied to determine the effect of APN treatment on the migration rate of the h‐JBMMSCs. The first was complete medium with or without APN (data not shown); the second was osteoblast‐inducing conditional medium with or without APN treatment for three times during a week, followed by scratching and assessment (data not shown); the third medium was the supernatant of the h‐JBMMSCs cultured in osteoblast‐inducing conditional medium with or without APN treatment for three times.

### Chemotaxis assay

Chemotaxis was measured using a transwell assay (PIEP 12R 48; Millipore, St. Louis, MO, USA). In the monoculture transwell assay, 1 × 10^5^ h‐JBMMSCs (200 μl) were planted into the upper compartment and 1.3 ml complete medium with or without APN or CXCL1 (Z03079, Genscript, USA) or CXCL8 (Z03138, Genscript, USA) was added into the lower compartment. In the co‐culture transwell assay, 1 × 10^5^ h‐JBMMSCs (1 ml) were placed into the lower compartment. After the h‐JBMMSCs reached confluence, osteoblast‐inducing conditional medium was added and the cells were cultured for 7 days, during which new medium and APN were added three times. Next, 1 × 10^5^ of the h‐JBMMSCs (200 μl) were placed into the upper compartment while ensuring that the volume of medium in the lower compartment was maintained at 1.3 ml. After 12 hrs, the upper compartment was washed with PBS three times and the cells were fixed with 10% paraformaldehyde for 10 min. The cells were then stained with crystal violet and photographed at 10× magnification (Nikon, Japan) after wiping out the cells from the upper surface of the upper compartment.

### RNA‐seq

h‐JBMMSCs were cultured in osteoblast‐inducing conditional medium for 7 days, during which new medium and APN were added three times. The cells were then washed with pre‐cooled PBS three times, and RNA was extracted using the TRIzol reagent (15596018; Ambion™, New York, NY, USA). The samples were then analysed using Illumina high‐throughput sequencing platforms (HiSeq™2500/Miseq™; Allwegene, Beijing, China).

### Real‐time PCR

mRNA was extracted from the cells using the TRIzol reagent (15596018; Ambion™, USA). The PrimeScript™ RT Master Mix (RR036A; Takara, Japan) was applied to generate cDNA. Relative quantitative PCR was then performed using SYBR (Roche Diagnostics, Basel, Switzerland) green master and the ABI 7500 real‐time PCR machine (Applied Biosystems, New York, NY, USA). The real‐time PCR was performed in 20 μl master mix containing 10 μl SYBR, 50 ng cDNA and 250 nM of each primer (CXCL1 forward primer—AACATGCCAGCCACTGTGAT, CXCL1 reverse primer—–AAAGGTAGCCCTTGTTTCCC; CXCL8 forward primer—CCACCGGAAGGAACCATCTC, CXCL8 reverse primer—TTCCTTGGGGTCCAGACAGA). The thermal cycling conditions were as follows: 10 min. at 95°C, 40 cycles at 95°C for 15 sec. and at 60°C for 60 sec. The relative expression levels of the genes were measured using the 2^−ΔΔCt^ method.

### ELISA

h‐JBMMSCs were cultured in osteoblast‐inducing conditional medium in 24‐well plates with or without APN. The cells in the APN‐treatment groups were cultured for 7 days with the addition of APN three times (once every 3 days). All the cells and supernatants were collected for ELISA. Total protein was extracted using the RIPA buffer (Applygen, Beijing, China), and the protein concentration was determined using the bicinchoninic acid reagent (Thermo Fisher Scientific, New York, NY, USA). ELISA was then used to assess the levels of CXCL1 (ab190805; Abcam, Cambridge, United Kingdom) and CXCL8 (ab174442; Abcam, Cambridge, United Kingdom) in the cytoplasm and supernatant according to the manufacturers’ instructions.

### Western blot

The h‐JBMMSCs were cultured in 60‐mm dishes, then starved for 12 hrs after reaching 90% confluence and treated with 1 μg/ml APN at the indicated time‐points. The primary antibodies used were as follows: p‐p38 MAPK (4511P; CST; USA) at 1:1000; p38 MAPK (8690P; CST; USA) at 1:1000; p‐AMPK (4186; CST; USA) at 1:1000; AMPK (5831; CST; USA) at 1:1000; and actin (g0314; Santa Cruz Biotechnology, Dallas, TX, USA) at 1:1000. Anti‐rabbit secondary antibody (7074S; CST; USA) was used at 1:5000. The protein concentration was detected using a chemiluminescence detection system (CW0048M; CWBIO, Beijing, China) and analysed using ImageJ software (National Institutes of Health, Bethesda, MD, USA).

### Statistical analysis

Data are represented as the mean ± standard deviation. Statistically significant differences (*P* < 0.05) between the groups were valued using one‐way analysis of variance or the Student's *t*‐test. All the statistical analyses were performed using SPSS 19.0 software (IBM Corp., Armonk, NY, USA).

## Results

### APN promoted the chemotaxis, but not the proliferation and migration, of h‐JBMMSCs

**Figure 1:**
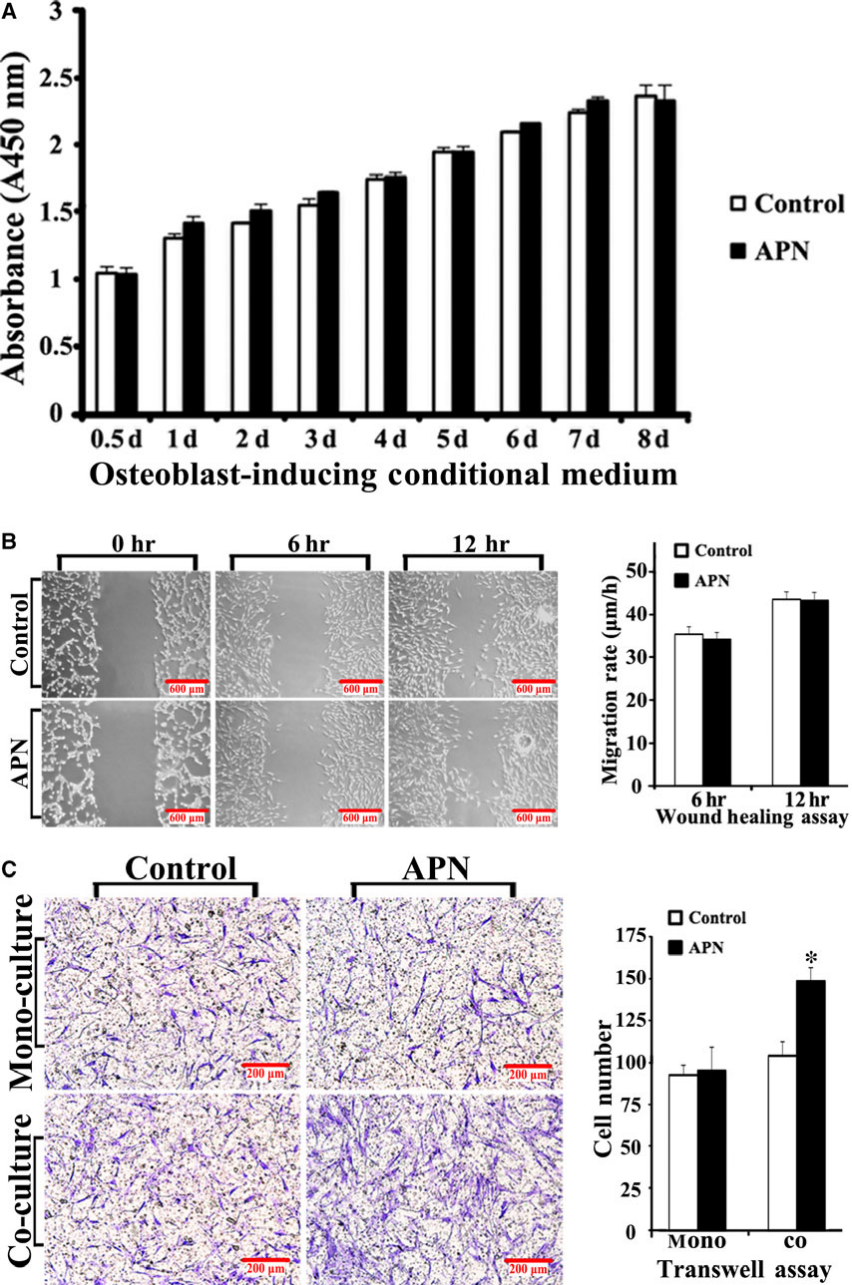
APN promoted the chemotaxis, but not the proliferation and migration, of h‐JBMMSCs. (**A**) h‐JBMMSCs were cultured in osteoblast‐inducing conditional media and treated with or without APN at 1 μg/ml once every 3 days. After 8 days of culturing in 96‐well plates, the cell proliferation was measured using the CCK‐8 assay at special time intervals. (**B**) The cell migration was evaluated by the wound healing assays at 0, 6 and 12 hrs after scratching. Three culturing media were used and described in detail in the materials and methods section. The migration rates are displayed as mean ± standard deviation (*n* = 3). (**C**) The chemotaxis was examined by the transwell assays. After 12 hrs, the cells in upper compartment were stained with crystal violet and photographed at 10× magnification. The cell number was displayed as mean ± standard deviation (*n* = 3; *P < 0.05).

The CCK‐8 cell proliferation assay showed that there was no significant difference between the control and the APN‐treatment group from day 1 to day 8 in the osteoblast‐inducing conditional medium (Fig. 1A; *P* > 0.05). The migration rates of the control and APN‐treatment group, as determined using the wound healing assay, were 35.33 ± 1.65 μm/h and 34.08 ± 1.77 μm/h, respectively, at 6 hrs and 43.33 ± 1.65 μm/h and 43.08 ± 1.89 μm/h, respectively, at 12 hrs (Fig. 1B; *P* > 0.05), with no significant difference. The monoculture transwell assay showed that the migrating cell counts of the h‐JBMMSCs did not significantly change after APN (1 μg/ml) treatment (Fig. 1C; 93 ± 6 *versus* 95 ± 14; *P* > 0.05) in the lower compartment. In the co‐culture transwell assay, after the h‐JBMMSCs in the lower compartment were treated with APN three times in osteoblast‐inducing conditional medium for 7 days, the upper compartment was seeded with h‐JBMMSCs from the lower compartment for 12 hrs. The migrating cell counts were 149 ± 11 and 104 ± 8 (Fig. 1C; *P* < 0.05) with and without APN treatment, respectively.

### RNA‐seq showed that APN treatment up‐regulated the chemotaxis‐related genes, CXCL1 and CXCL8

**Figure 2:**
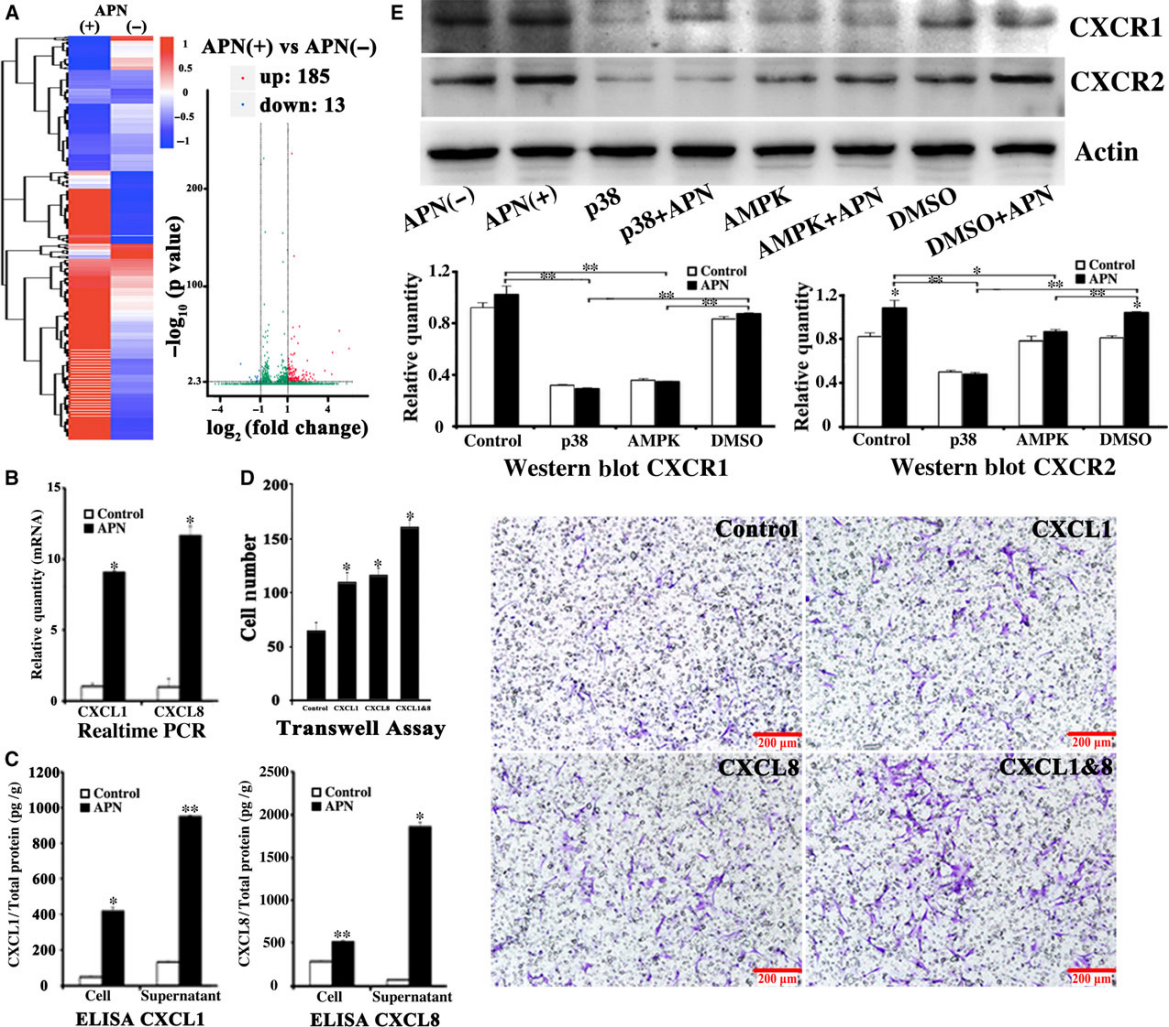
APN promoted h‐JBMMSCs up‐regulating CXCL1 and CXCL8. (**A**) h‐JBMMSCs were cultured in osteoblast‐inducing conditional media with or without APN (1 μg/ml) for 1 week, and the total RNA was analysed using RNA‐seq. A total of 198 genes showing differential expression were identified, including 185 up‐regulated and 13 down‐regulated genes in the APN‐treatment group (*P* < 0.005). The CXCL1 and CXCL8 were selected as the target genes. To validate the results of RNA‐seq, real‐time PCR (**B**) and (**C**) ELISA were used to measure the expression of CXCL1 and CXCL8 in the control and APN‐treated groups (*n* = 3; *P < 0.05; **P < 0.01). (**D**) The exogenous CXCL1 or CXCL8 proteins were added in the lower compartment to verify its chemotaxis effect. The cell number was displayed as mean ± standard deviation (*n* = 3; *P < 0.05). (**E**) The Western blot was used to evaluate the expression of CXCR1 and CXCR2, the specific receptor of CXCL1 and CXCL8, after different treatments (*n* = 3; *P < 0.05; **P < 0.01).

h‐JBMMSCs were cultured in osteoblast‐inducing differentiation media for 1 week, and the total RNA extracted from the control group and APN‐treatment group was analysed using RNA‐seq. A total of 198 genes showing differential expression were identified, including 185 up‐regulated and 13 down‐regulated genes in the APN‐treatment group (*P* < 0.005). The fold change in the expression of CXCL1 and CXCL8 was 4.8 and 5.5, respectively, in the APN‐treatment group compared to the expression in the control group (Fig. 2A).

### APN promoted h‐JBMMSCs chemotaxis through CXCL1 and CXCL8

**Figure 3:**
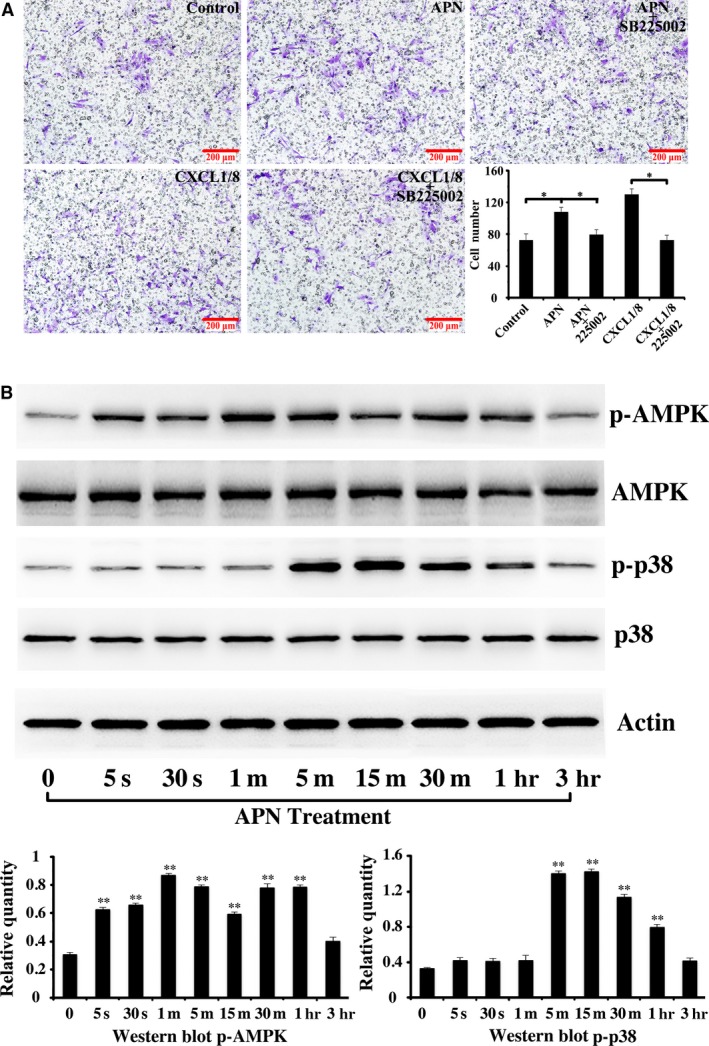
APN promoted h‐JBMMSCs chemotaxis through CXCL1/8 and activated the phosphorylation of AMPK and p38 MAPK in a time‐dependent manner. (**A**) SB225002 could inhibited h‐JBMMSCs chemotaxis in both the co‐culture and the monoculture transwell systems (*n* = 3; *P < 0.05). (**B**) The h‐JBMMSCs were cultured in 60‐mm dishes, then starved for 12 hrs after reaching 90% confluence and treated with 1 μg/ml APN at the indicated time‐points. Then, phospho‐p38 MAPK, total p38 MAPK, phospho‐AMPK, total AMPK and actin were examined by Western blotting. The protein concentration was also analysed using Image J software (*n* = 3; **P < 0.01).

To validate the results of RNA‐seq, real‐time PCR and ELISA were used to measure the expression of CXCL1 and CXCL8 in the control and APN‐treatment groups. After culturing the cells in osteoblast‐inducing differentiation medium for 1 week, real‐time PCR showed that the mRNA level of CXCL1 and CXCL8 increased 9.1 times (control: 1.03 ± 0.20 *versus* APN treatment: 9.09 ± 0.05; *P* < 0.05) and 11.7 times (control: 1.00 ± 0.63 *versus* APN treatment: 11.70 ± 0.63; *P* < 0.05), respectively, in the APN‐treatment group (Fig. 2B). ELISA showed that the CXCL1/total protein (pg/g) level in the cells and supernatants was increased 8.9 times (control: 47.82 ± 3.54 *versus* APN treatment: 419.74 ± 17.80; *P* < 0.05) and 7.1 times (control: 133.09 ± 3.62 *versus* APN treatment: 949.49 ± 7.15; *P* < 0.01), respectively, in the APN‐treatment group than in the control group. The CXCL8/total protein (pg/g) level of the cells and supernatants was increased 1.8 times (control: 281.92 ± 11.21 *versus* APN treatment: 514.52 ± 14.52; *P* < 0.01) and 34.8 times (control: 70.49 ± 1.33 *versus* APN treatment: 1859.11 ± 53.44; *P* < 0.05), respectively, in the APN‐treatment group (Fig. 2C). In the monoculture transwell assay, the upper compartment was seeded with h‐JBMMSCs and the lower compartment was treated with PBS (63 ± 11) or CXCL1 (112 ± 13) or CXCL8 (122 ± 11) or CXCL1/CXCL8 (162 ± 13), respectively. The migration of the cells in the PBS treatment group was less than that of the cells in the other three groups (Fig. 2D; *P* < 0.05). Western blot revealed that h‐JBMMSCs expressed CXCR1 and CXCR2 and that CXCR2 was up‐regulated with APN treatment (Fig. 2E; *P* < 0.05). When h‐JBMMSCs were pre‐treated with SB225002, the migrating cells were inhibited in both the co‐culture (control: 73 ± 8 *versus* APN: 108 ± 6, *P* < 0.05; APN: 108 ± 6 *versus* APN + SB225002: 80 ± 6, *P* < 0.05; control: 73 ± 8 *versus* APN + SB225002: 80 ± 6, *P* > 0.05) and the monoculture (CXCL1/8: 130 ± 7 *versus* CXCL1/8 + SB225002: 73 ± 6, *P* < 0.05) transwell systems (Fig. 3A).

### APN activated the phosphorylation of AMPK and p38 in a time‐dependent manner

Western blot analysis showed that AMPK phosphorylation significantly increased in response to APN treatment over time from 5 sec. to 60 min. (*P* < 0.01; Fig. 3B) and p38 MAPK phosphorylation increased significantly from 5 min. to 60 min. (*P* < 0.01; Fig. 3B).

### WZ4003 and SB203580 inhibited the APN‐induced overexpression of CXCL1 and CXCL8 and h‐JBMMSC chemotaxis

**Figure 4:**
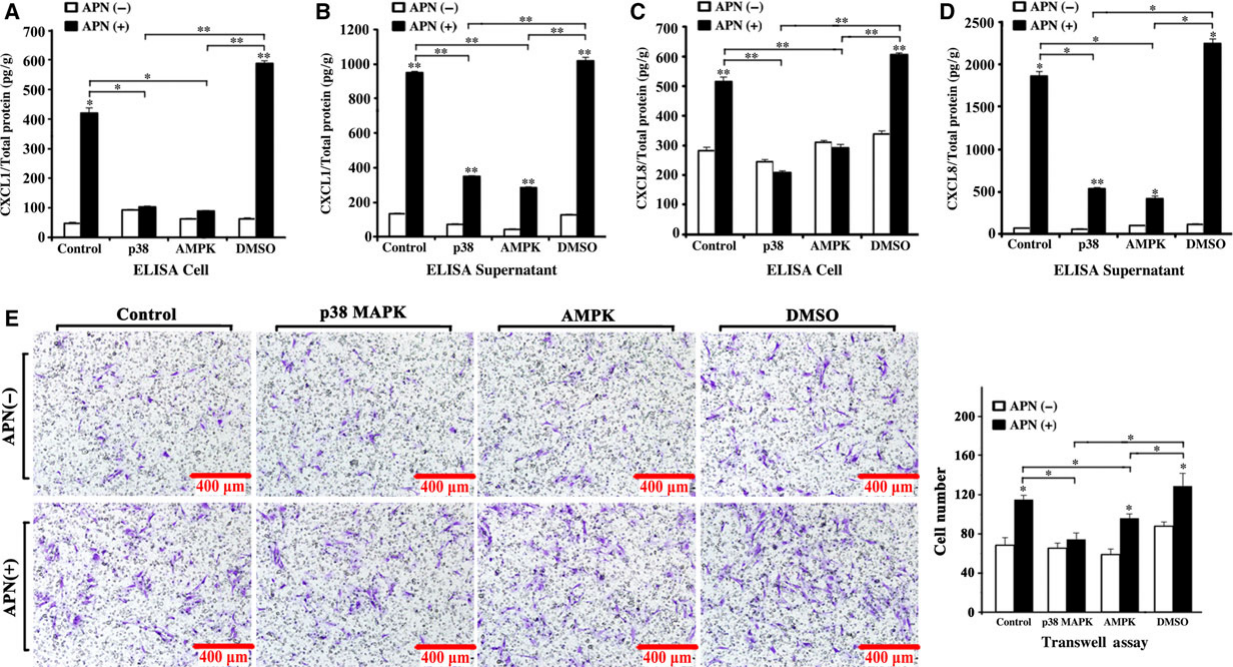
The AMPK inhibitor, WZ4003, and the p38 MAPK inhibitor, SB203580, could inhibit the APN‐induced overexpression of CXCL1 and CXCL8 and h‐JBMMSCs chemotaxis. After treated with WZ4003 and SB203580, the expression of CXCL1 (**A** and **B**) and CXCL8 (**C** and **D**) in the cells and supernatants of h‐JBMMSCs treated with or without APN was evaluated by the ELISA assay (*n* = 3; *P < 0.05; **P < 0.01). (**E**) After treated with WZ4003 and SB203580, the chmotaxis of h‐JBMMSCs treated with or without APN was evaluated by the transwell assay (*n* = 3; *P < 0.05).

The AMPK inhibitor, WZ4003, and the p38 MAPK inhibitor, SB203580, could suppress the APN‐induced increase in the CXCL1/total protein and CXCL8/total protein levels in the cells (Fig. 4A,C; *P* > 0.05) but could not suppress the APN‐induced increase in the CXCL1/total protein and CXCL8/total protein levels in the supernatants (Fig. 4B,D; *P* < 0.01). Compared to the control‐APN(+) group, the p38 MAPK‐APN(+) group and AMPK‐APN(+) group showed significantly lower levels of CXCL1/total protein and CXCL8/total protein in the cells and the supernatants (Fig 4. A, B, C, D; *P* < 0.05). Treatment with the inhibitors significantly down‐regulated CXCR1 and CXCR2 (*P* < 0.05), but showed no response to APN treatment (Fig. 2E). In the co‐culture transwell assay, all the groups, except the p38 MAPK group, showed a higher number of directly migrating APN(+) cells than of APN(−) cells. The number of directly migrating cells was significantly higher in the control‐APN(+) group than in the p38 MAPK‐APN(+)and AMPK‐APN(+) groups (Fig. 4E; *P* < 0.05).

## Discussion

In the current study, we focused on determining whether APN can trigger the chemotaxis of h‐JBMMSCs and on identifying the intracellular mechanisms and chemoattractants involved in such APN‐induced recruitment of h‐JBMMSCs. To evaluate the biological functions of APN, we conducted the CCK‐8 assay, wound healing assay and transwell assay to evaluate the effect of APN treatment on the proliferation, migration and chemotaxis of h‐JBMMSCs. It must be emphasized here that the APN used in the study was produced using HEK 293 cells to exclude the influence of endotoxin stimulus (<0.01 EU/μg, as determined using the LAL method). The proliferation rate of the h‐JBMMSCs after APN treatment (1 μg/ml) in either complete medium (data not shown) or osteoblast‐inducing conditional medium showed no significant difference compared with the untreated group (Fig. 1A), which was consistent with the report of the study conducted by Williams *et al*. 26. In the wound healing assay, we used three culturing media (as described in the materials and methods section). As shown in Fig. 1B, the h‐JBMMSCs showed only random migration and no statistically significant difference was observed between the APN‐treated and the untreated group at 6 hrs or 12 hrs. This result indicated that APN possibly had no effect on the proliferation and migration of h‐JBMMSCs. Chemotaxis describes the ability of cells to sense the gradient of an extracellular factor and directly migrate along the gradient, which is classically studied *in vitro* using transwell assays. We also conducted transwell assays in our study and found no significant difference between the control and APN‐treated group after APN was added in the lower compartment, which excluded the possibility of APN affecting the direct chemotaxis of the h‐JBMMSCs. Interestingly, when we added the supernatant of the h‐JBMMSCs stimulated with APN for 1 week into the lower compartment, the chemotaxis of the h‐JBMMSCs greatly increased in comparison with that of the control group. The reason for this may be that some soluble factors capable of activating the chemotaxis were secreted from the h‐JBMMSCs after treatment with APN. Therefore, next, we thought it essential to analyse the contents of the supernatant after APN treatment in the subsequent experiments.

To identify the chemokines involved in the APN‐induced chemotaxis of h‐JBMMSCs, after culturing the cells in osteoblast‐inducing conditional medium with or without APN for 1 week, the total RNA of the h‐JBMMSCs was extracted and analysed using RNA‐seq. Among the up‐regulated genes, CXCL1 and CXCL8 genes were selected as the target genes, and the fold changes in their expression levels were 4.8 and 5.5, respectively, between the APN‐treated and untreated groups (Fig. 2A; *P* < 0.01). Real‐time PCR (Fig. 2B) and ELISA (Fig. 2C) were further used to validate the results of the RNA‐seq, which demonstrated that the expression level of CXCL1 and CXCL8 in the APN‐treated group was significantly increased both at the RNA and at the protein level. Moreover, after stimulating with exogenous CXCL1 or CXCL8 proteins, more cells migrated through the polycarbonate membrane than the control group (Fig. 2D). In addition, the specific receptor of CXCL1 and CXCL8, CXCR2, was up‐regulated upon APN treatment (Fig. 2E; *P* < 0.05). When h‐JBMMSCs were pre‐treated with SB225002, the APN‐induced chemotaxis and CXCL1/8‐induced chemotaxis were inhibited. Other reports also proved that SB225002 could inhibit the CXCL1/8‐induced chemotaxis in neutrophils 27. Based on these results, we concluded that APN could promote the migration of h‐JBMMSCs through CXCL1 and CXCL8.

Next, we needed to determine the mechanism underlying the APN‐driven chemotaxis during the osteogenesis of h‐JBMMSCs. APN has already been reported to activate the adenosine monophosphate‐activated protein kinase (AMPK) signalling pathway by binding with the membrane receptor AdipoR1 28. In addition, the results from our present study confirmed that APN could promote the osteogenesis of h‐JBMMSCs through the APPL1‐p38 MAPK signalling pathway 24. The AMPK and p38 MAPK pathways are both important signalling nodes capable of regulating cellular metabolism and participate in various life processes. Although AMPK and p38 MAPK have been established as downstream effectors of APN receptor activation, their possible role in the regulation of chemotaxis has not been clarified so far. In our study, we found that the phosphorylation of AMPK and p38 was activated in a time‐dependent manner upon APN treatment (Fig. 3B). Treatment with the inhibitors of AMPK and p38 MAPK (Fig. 4A) separately suppressed the APN‐induced CXCL1/total protein and CXCL8/total protein levels in the cells, which indicated that the APN‐induced increase in the levels of CXCL1 and CXCL8 was through the AMPK and p38 MAPK signalling pathway. When the cells were cultured with the supernatant, although we added the inhibitors, the levels of CXCL1/total protein and CXCL8/total protein were not suppressed, which suggested the involvement of other pathways in the modulation of chemotaxis. The CXCL1 and CXCL8 receptors were all down‐regulated when the AMPK and p38 MAPK inhibitors were added. The same trend (Fig. 4B) was also observed in the transwell assay. In summary, our results indicated that APN could promote CXCL1 and CXCL8 expression through the AMPK and p38 MAPK signalling pathway. However, other underlying pathways might also be involved in the APN‐induced modulation of chemotaxis and need to be explored in detail in the future.

**Figure 5:**
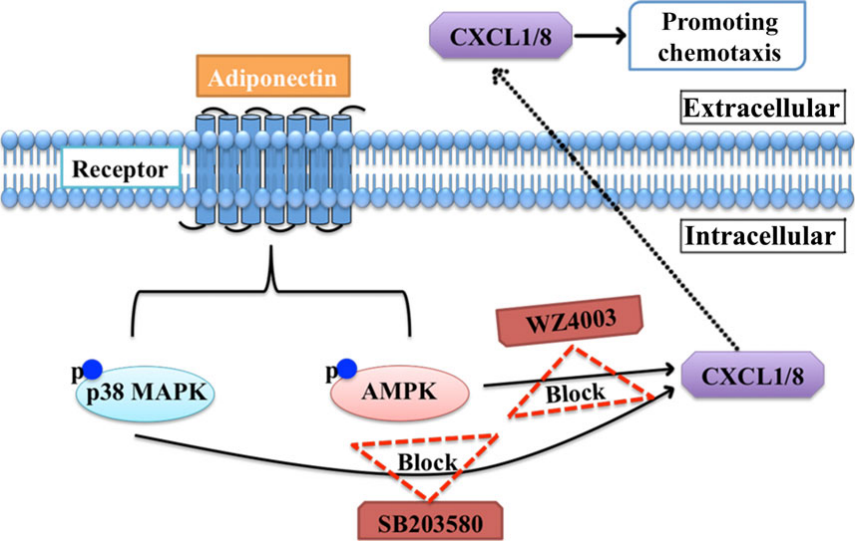
Schematic of APN up‐regulating CXCL1/8 *via* promoting phosphorylation of AMPK and p38 MAPK in h‐JBMMSCs. APN increases chemotaxis of h‐JBMMSCs through APN‐AMPK/p38 MAPK‐CXCL1/8 pathway. The APN‐induced CXCL1/8 up‐regulation could be blocked by AMPK inhibitor, WZ4003, and the p38 MAPK inhibitor, SB203580.

To the best of our knowledge, this is the first study showing the effect of APN on the chemotaxis of h‐JBMMSCs during their differentiation into the osteoblastic lineage. The involved AMPK and p38 MAPK pathways may contribute to our understanding of the mechanism by which APN regulated the chemotaxis of h‐JBMMSCs during osteogenesis. However, the mechanisms of APN‐induced osteogenesis are complicated and need detailed investigation in future studies. In conclusion, the results of our present study indicate that APN‐induced osteogenesis might involve two synchronous and independent steps: promotion of the chemotaxis of h‐JBMMSCs to the extraction sockets and facilitation of the osteogenic differentiation of h‐JBMMSCs (Fig. 5). This study provides new insights on the role of APN in bone formation and provides evidence indicating the potential of the clinical application of APN in tissue engineering.

## Conflicts of interest

The authors declare no conflict of interest.
